# In situ fibrillizing amyloid-beta 1-42 induces neurite degeneration and apoptosis of differentiated SH-SY5Y cells

**DOI:** 10.1371/journal.pone.0186636

**Published:** 2017-10-24

**Authors:** Jekaterina Krishtal, Olga Bragina, Kristel Metsla, Peep Palumaa, Vello Tõugu

**Affiliations:** Department of Chemistry and Biotechnology, Tallinn University of Technology, Tallinn, Estonia; Nathan S Kline Institute, UNITED STATES

## Abstract

The progression of Alzheimer’s disease is causatively linked to the accumulation of amyloid-β aggregates in the brain, however, it is not clear how the amyloid aggregates initiate the death of neuronal cells. The *in vitro* toxic effects of amyloid peptides are most commonly examined using the human neuroblastoma derived SH-SY5Y cell line and here we show that differentiated neuron-like SH-SY5Y cells are more sensitive to amyloid peptides than non-differentiated cells, because the latter lack long neurites. Exogenous soluble amyloid-β 1–42 covered cell bodies and whole neurites in differentiated cells with dense fibrils, causing neurite beading and fragmentation, whereas preformed amyloid-β 1–42 fibrils had no toxic effects. Importantly, spontaneously fibrillizing amyloid-β 1–42 peptide exhibited substantially higher cellular toxicity than amyloid-β 1–40, which did not form fibrils under the experimental conditions. These results support the hypothesis that peptide toxicity is related to the active fibrillization process in the incubation mixture.

## Introduction

Alzheimer’s disease (AD), a complex neurodegenerative disorder, is the most prevalent cause of dementia worldwide. Although the disease was first described more than 100 years ago, the etiology of AD is still elusive. Amyloid plaques in the patient’s brain are the primary hallmark of AD and the evidence for the central role of amyloid beta (Aβ) peptides–the main component of amyloid plaques- in the pathogenesis of AD is very strong [1, 2]. For more than twenty years, the amyloid cascade hypothesis has served as the dominant framework for AD studies, however, a clear understanding and description of the molecular events leading to neurodegeneration is still missing and several alternative explanations for disease progression are under discussion [3–6]. It has been shown that various aggregated forms of Aβ peptides are neurotoxic in animal models, primary neuronal cultures and immortalized cell lines [7–9]. However, the results of Aβ toxicity studies are often controversial and have not yet provided a clear understanding of the disease mechanism or the molecular events underlying Aβ toxicity. Since mainly neuronal cells die during neurodegeneration, it is likely that Aβ acts via a specific mechanism to induce neuronal cell death. Previous studies on primary neurons have shown that Aβ causes neuritic abnormalities in neuronal cultures [10, 11], which are also initial signs of dying neurons in AD. Therefore, it is important to use relevant cellular models for the study of the neuron-specific effects of Aβ peptides. The human SH-SY5Y cell line is widely used as a model for different neurodegenerative diseases including AD [12]. The phenotype of SH-SY5Y cells can be manipulated by inducing different programs of neural differentiation, however, in most (81.5%) publications non-differentiated cells are used [12]. Due to their dopaminergic character, SH-SY5Y cells are generally considered as a model for Parkinson’s disease, however, they can be differentiated to dominantly cholinergic phenotype suitable for AD studies by treatment with retinoic acid (RA) and brain-derived neurotrophic factor (BDNF) [13]. Aβ toxicity on SH-SY5Y cells has been determined in a large number of studies, however, there are only a few examples examining Aβ-induced toxicity in SH-SY5Y cells where cell proliferation has been suppressed and preliminary differentiation initiated by RA [14–16]. Additionally to the best of our knowledge, there are currently no available data investigating whether Aβ is toxic for RA/BDNF differentiated SH-SY5Y cells.

Another important yet understudied area within the framework of the amyloid hypothesis concerns the exact nature of the toxic form(s) of Aβ. In the AD brain, the “extra” amyloid in developing plaques is in the form of amyloid fibrils. The fibrillation is an autocatalytic process—once the fibrils are formed they start to grow by trapping monomers. Due to the relatively low toxicity of Aβ monomers and preformed Aβ fibrils for cell cultures, the pathogenic entities of the peptide are intensively searched for and the toxic effects have been attributed to a wide variety of species, including oligomers, intermediate aggregates and peptide-copper complexes [17–20]. In many cases the peptide formulations have been pretreated in conditions entirely different from those that can occur in living organisms. For instance, a popular oligomerization procedure involves fast dilution of concentrated peptide solutions in an organic solvent to form a supersaturated solution [21, 22]. In 1994 Lambert and colleagues demonstrated the toxic effect of Aβ42 on RA pretreated SH-SY5Y cells and attributed this effect to the peptide oligomers (DMSO-induced) [23]. Recent studies have demonstrated that the toxic entities of the peptide can be the metastable particles that form during the natural fibrillization of Aβ [20, 24], and serve to highlight that these more natural fibrils should be preferred over artificially generated oligomers.

Here we used RA and BDNF differentiated human neuroblastoma SH-SY5Y cells, a simple model suggested for neuronal screening [25–27], to study the effects of Aβ-peptides. The differentiation of SH-SY5Y cells increased their susceptibility to Aβ and allowed the description and quantification of pathological changes associated with primary neuronal cultures and patients with AD [28]. The obtained results support the hypothesis that neuron-specific Aβ toxicity may be caused by the intermediate amyloid aggregates that form during the fibrillization of Aβ-peptides [29]. In our opinion, further study of the differentiated SH-SY5Y cells will aid our understanding of the molecular mechanisms responsible for the pathological processes induced by amyloid peptides in cells of human origin.

## Materials and methods

### Chemicals and reagents

Cell culture associated reagents were purchased from Gibco, Thermo Fisher: Dulbecco's Modified Eagle's Medium (DMEM), 0.25% Trypsin-EDTA solution. Penicillin/Streptomycin solution was from PAA, Cambridge, UK. Brain derived neurotrophic factor was obtained from Alomone Labs, Jerusalem, Israel. Recombinant human Aβ40 and Aβ42 peptides (TFA salts, purity >97%) were from rPeptide, Bogart, GA 30622, USA. Tween-20 (Ferak, Berlin, Germany) WST-1 cell viability assay was purchased from Roche, Switzerland, and the Caspase-Glo assay kit from Promega Co, Madison, WI, USA. 1,1,1,3,3,3-hexafluoro-2-propanol (HFIP), 4-(2-hydroxyethyl)-1-piperazineethanesulfonic acid (HEPES), poly-L-lysine, retinoic acid, CalceinAM (calcein-acetoxymethyl ester), sodium chloride; goat serum; staurosporine, 4',6-diamidino-2-phenylindole (DAPI), PBS were obtained from Sigma Aldrich.

Antibodies against a microtubule component βIII-tubulin TUJ-1 were obtained from Abcam, Cambridge, UK; anti-APP/Aβ antibody—from Millipore, Darmstadt, Germany. Guinea pig anti-Synaptophysin1 was from Synaptic Systems, Goettingen, Germany. Secondary antibodies were Alexa-488 and Alexa-568 from Invitrogen.

### Cell culture

SH-SY5Y cells (ATCC, VA 20110, USA) were cultured in DMEM without Phenol Red and supplemented with 10% FBS and 1X Penicillin/Streptomycin solution at 37°C and 5% CO_2_, to allow fluorescence measurements (Gibco, Thermo Fisher). The medium was changed every 2–3 days and cells were split using 0.25% Trypsin-EDTA solution.

### SH-SY5Y differentiation

A differentiation protocol from Ref. [25, 26] with several changes was applied. The cells were seeded onto microtiter plates (Greiner Bio-one) coated with poly-L-lysine to allow neurite outgrowth and differentiation. Cells were grown for 1 day prior to differentiation. The next day, 10 μM RA in DMEM with 10% FBS was applied; the medium with RA was changed every day. After a 4-day incubation with RA, BDNF at the final concentration of 50 ng/ml was applied in DMEM without serum for 2 days. After 6 days of differentiation the cells were used for the experiments. (S1 Fig).

### Preparation of amyloid-β peptide solutions

Lyophilized Aβ40 and Aβ42 peptides were dissolved in HFIP to get a homogeneously monomeric preparation, vortexed briefly and incubated for 1 hour at room temperature. Next, defibrillized peptide solutions were aliquoted and dried in a vacuum desiccator overnight. Peptide aliquots were stored at -80°C until usage. Peptide quality was assessed by ^1^H NMR, MALDI-MS and SDS–PAGE (S2 Fig). For experiments, Aβ40 and Aβ42 aliquots were dissolved in 20 mM HEPES buffer containing 100 mM NaCl at pH 7.3 to the final concentration of 160 μM and vortexed for 10 sec. The prepared peptide solution was immediately applied to the serum free cell culture. Preformed fibrils were prepared as described in Ref. [30].

### Evaluation of neurite degeneration

Cells were grown in 6 cm cell culture plates on 47 mm glass coverslips coated with poly-L-lysine and differentiated as described above. Photomicrographs of at least 12 random areas of neurites were taken using a Zeiss Duo 510 META microscope with a 20X objective or a 63X objective with oil immersion. Neurites were stained with CalceinAM for the analysis. General morphology of neurites was obtained with the differential interference contrast (aperture DIC2) technique. All microscopy experiments were performed in an incubation chamber at 37°C in the presence of 5% CO_2_.

Photomicrographs of neurites in randomly selected areas were stored and processed using LSM Image Browser software. The method to evaluate neurite degeneration was adopted from Ref. [31] with small modifications. The number of beads per total length of measured neurites was counted. Medium or thin neurites in captured regions were chosen for this purpose. The number of beadings/50 μm length was counted and averaged over at least 8 neurites for each area. The proportion of fragmented neurites was counted for each area and expressed as a percentage of the total amount of counted neurites longer than 100 μm (S3 Fig). The procedures were repeated on three different samples (vehicle, Aβ40 and Aβ42). The results of three independent experiments were averaged and presented with SEM without normalization. The photomicrographs were coded for the evaluator by random codes.

### Immunofluorescence

The neuronal phenotype of differentiated SH-SY5Y was established by immunocytochemical staining with antibodies against a microtubule component βIII-tubulin TUJ-1 (1:2000). For the determination of Aβ location in the cell culture, the cells were stained with anti-APP/Aβ antibody (1:2000) and guinea pig anti-Synaptophysin1 (1:2000). Samples were fixed for 15 min at 4°C in methanol (for microtubule visualization) or 4% paraformaldehyde (anti-APP/Aβ staining). Blocking was performed with 3% goat serum. The samples were washed with PBS to remove excess protein before incubation with primary monoclonal antibodies in 0.25% Tween-20 solution 1:2000 at 4°C overnight. Samples were washed with PBS for 5 min before incubation with secondary Alexa-488 or Alexa-568 conjugated goat anti-mouse antibody 1:2000 or goat anti-guinea pig antibody 1:2000 in PBS for 1 hour at room temperature. Nuclei were stained with DAPI for 5 min at room temperature. Cells were investigated using a confocal Zeiss Duo 510 META microscope with a 20X objective or a 63X objective with Zeiss oil immersion.

### Cell cytotoxicity assays

The effects of peptides on the cells were determined using the WST-1 cell viability assay and the membrane permeability assay with propidium iodide (PI). 20 mM HEPES buffer containing 100 mM NaCl at pH 7.3 was used as a negative control (hereafter Vehicle) added to serum-free medium in equal amounts with the peptide sample. 5 μl/well of WST-1 reagent was added to 100 μl cell culture medium, incubated at 37°C for 2 h and the absorbance measured at 450 nm. PI in PBS (0.5 mM) was added to 100 μl cell culture 1.5 μl/well and incubated for 2 h at 37°C. Fluorescence was measured using a TECAN Genios Pro microplate reader (Tecan, Switzerland) (excitation 540 nm, emission 612 nm). Data from at least three independent experiments, all experimental points in triplicates, were normalized taking Vehicle as 100%.

### Measurement of caspase activity

The differentiated SH-SY5Y cells were plated in 96-well plates. The activity of caspase-3/7 was measured using a Caspase-Glo assay kit. In this kit a substrate for luciferase is released when the colorimetric substrate, containing the tetrapeptide sequence DEVD, is cleaved by caspase-3/7. After cell treatment with the buffer (the vehicle), staurosporine or Aβ (20 μM), Caspase Glo-3/7 reagent was added to the culture medium and incubated at room temperature for 1 h. The intensity of the chemiluminescence was measured using a TECAN Genios Pro microplate reader.

### Statistical analysis

The differential significance of the results obtained was determined by one-way ANOVA followed by a Bonferroni's multiple comparisons test at the 0.05 level. All values are presented with means ± SEM, except where otherwise indicated. Raw data values can be seen in S1–S7 Tables. The number of experiments is represented by *n*. p-values of post-hoc test are denoted by asterisks (* p≤0.01;** p≤0.005). Statistical analysis was carried out using GraphPad Prism 6.

## Results

### RA/BDNF differentiation increases the susceptibility of SH-SY5Y cells towards Aβ toxicity

**Fig 1:**
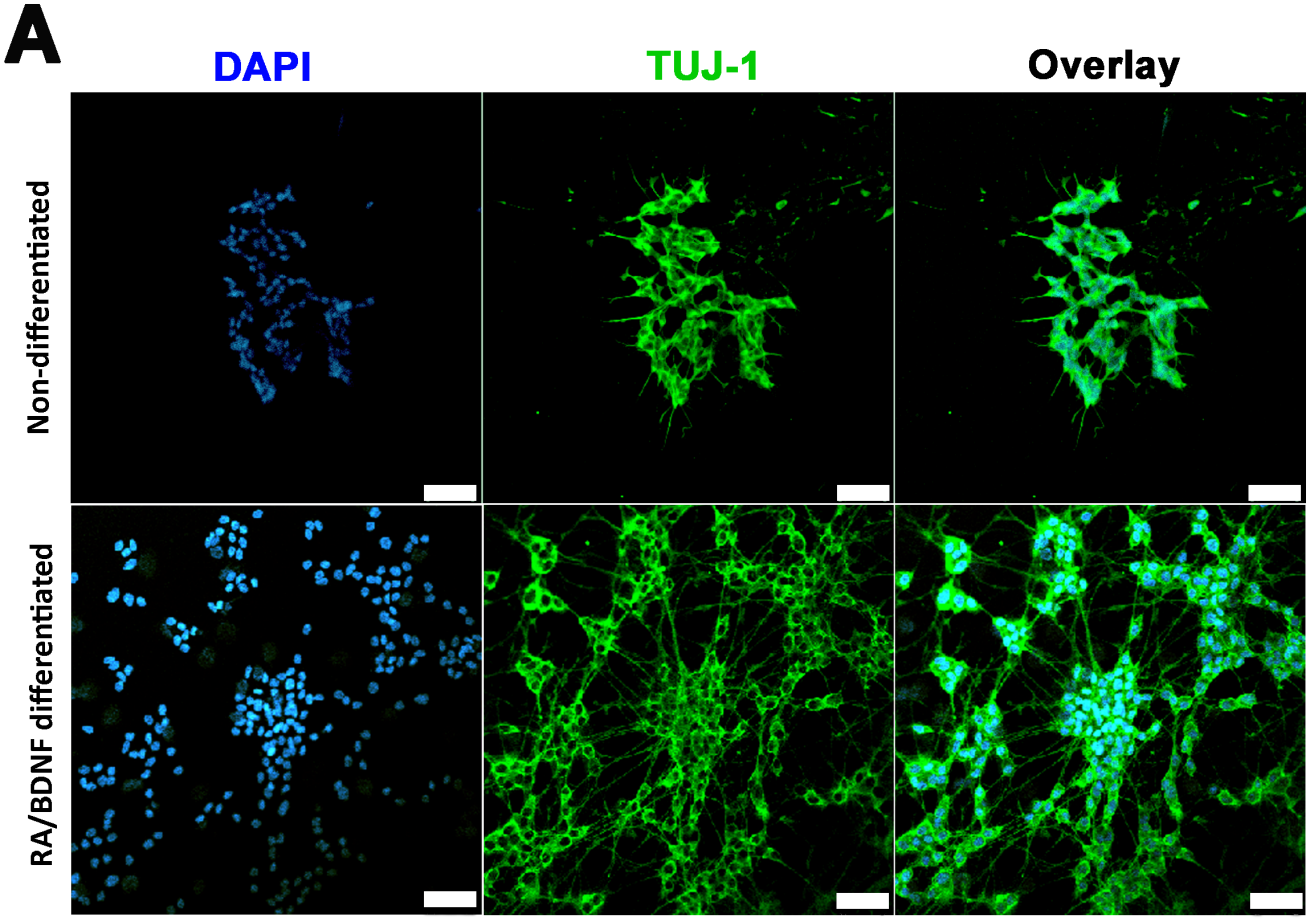
RA/BDNF differentiated cells establish a neuron-like phenotype with long neurites. (A) Immunocytochemistry of non-differentiated and RA/BDNF differentiated SH-SY5Y cells for DAPI (blue; left), anti-TUJ-1 (green; middle). Scale bar 50 μm.

Neuron-like SH-SY5Y human neuroblastoma cells were generated using a differentiation procedure modified from previously descried protocols [25–27]. The cells were pre-treated for 4 days with 10 μM *all-trans* RA to induce the expression of TrkB receptors and increase their biological responsiveness to neurotrophic factor treatment [32]. After the sequential treatment with RA and BDNF, the cells developed long beta III tubulin (TUJ-1)-positive neurites that formed networks characteristic to neurons (Fig 1A).

**Fig 2:**
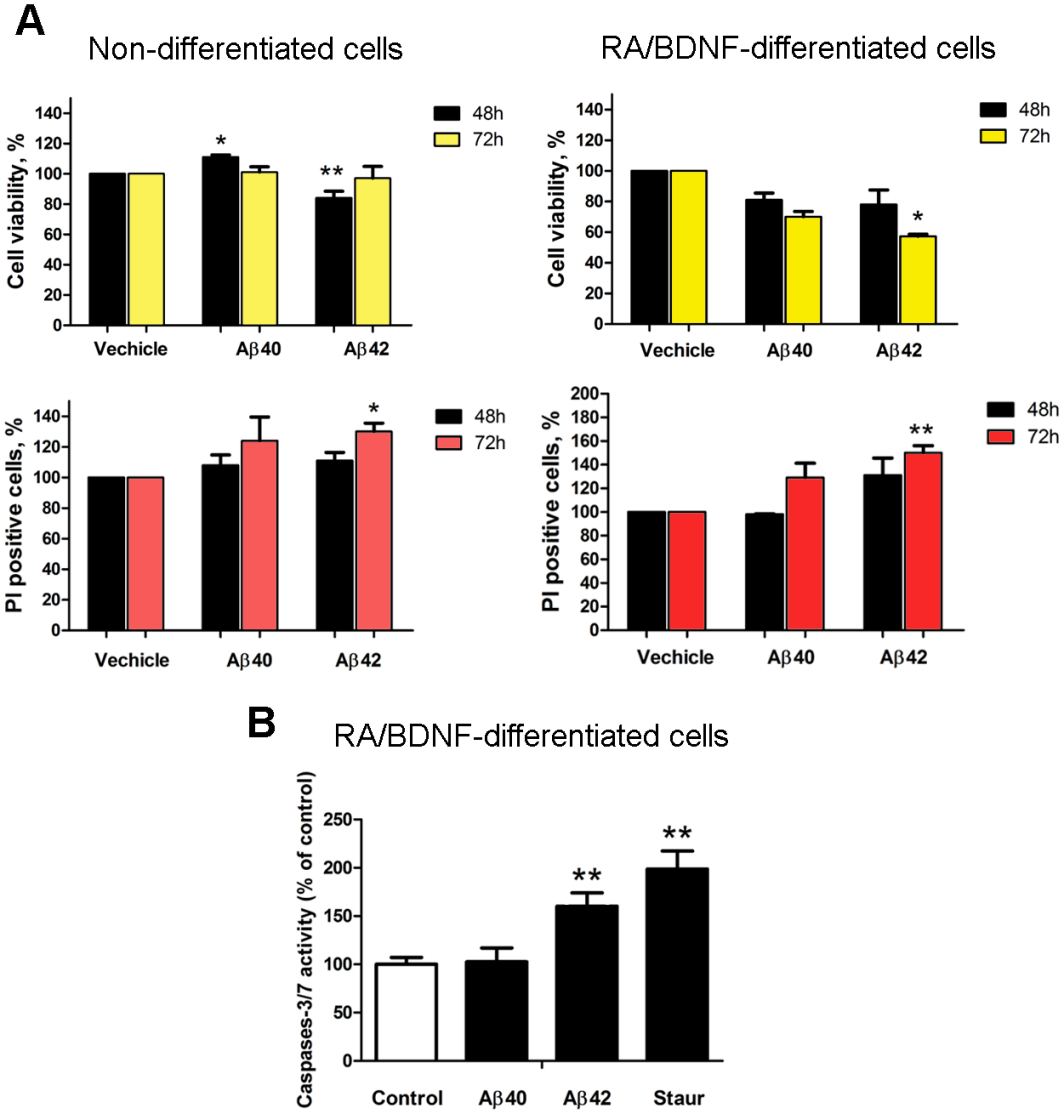
Aβ reduces viability and activates caspases-3/7 in differentiated SH-SY5Y cells. (A) Cell viability was measured with the WST-1 test and membrane integrity was measured using propidium iodide 48h and 72h after incubation with 20μM peptides. The figure displays the mean± SEM; at least *n = 3* independent experiments in case of Aβ42 and *n = 5* experiments in case of Aβ40; **p≤0.005; *p≤0.01; (B) Effect of Aβ42 on the activity of caspase-3 and/or 7. Caspase activity was determined by measuring DEVD-AFC hydrolysis in lysates from SH-SY5Y cells treated with 20μM Aβ42 for 48h. The figure displays the mean ± SD; *n = 5*; **p≤0.005. One-way ANOVA followed by a Bonferroni's multiple comparisons test at the 0.05 level was used to determine the difference between the conditions.

Aβ-peptides had a small effect on the viability of undifferentiated SH-SY5Y cells in serum-free DMEM. The influence of serum withdrawal on cell viability during 3 days was not significant (S4 Fig). In the presence of Aβ42 the cell viability decreased to 84 ± 5%, after a 48-hour incubation, whereas Aβ40 tended to increase the viability (111 ± 2%) (Fig 2A, left panels; S1 and S2 Tables). No changes in cell viability according to the WST-1 test were detected after a 72 h incubation with either peptide at a concentration of 20 μM. At the same time, the membrane permeability test with PI showed a statistically significant increase in cell death after a 72 h incubation with Aβ42 (130 ± 6%). Aβ40 also increased cell death, however, the results varied remarkably between independent repeats (124 ± 16%) and the effect was statistically insignificant. Thus, both the cell viability and the membrane permeability tests showed that the toxic influence of Aβ-peptides on non-differentiated SH-SY5Y cells is relatively small. Aβ-peptides could eventually affect a small subpopulation of non-differentiated SH-SY5Y cells (S5 Fig), however the majority of the cells continued to proliferate, which complicates the interpretation of viability measurements. Nevertheless, the non-differentiated SH-SY5Y cells with a low sensitivity to Aβ and heterogeneous RA-differentiated cells cannot be used as a reliable model for the study of the toxic effects of native Aβ.

Aβ-peptides were more toxic to the RA/BDNF neuronally differentiated SH-SY5Y cells according to both the WST-1 and the PI tests (Fig 2A, right panels; S3 and S4 Tables). In the WST-1 test, cell viability started to decline after a 48-hour incubation (81 ± 5%for Aβ40 and 78 ± 9% for Aβ42), however, statistically significant effects (70 ± 3% and to 57.3 ± 1.3%) in the presence of Aβ40 and Aβ42, respectively, were observed after 72 h. A statistically significant increase in the membrane permeability was observed after 72 h only in the case of Aβ42 (150 ± 6%). The PI fluorescence also slightly increased after a 72 h incubation with Aβ40 (129 ± 12%) and after 48 h with Aβ42 (131 ± 15%). The neuron-like RA/BDNF differentiated SH-SY5Y cells appeared to be more susceptible to Aβ-peptides than the non-differentiated SH-SY5Y cells.

### Aβ42, but not Aβ40, induces apoptosis of differentiated SH-SY5Y

To prove the apoptotic nature of Aβ-induced cell death, we examined the activation of caspase-3/7. After a 48 h incubation, Aβ42 significantly increased caspase-3/7 activity (160±14%) compared to that of the vehicle (Fig 2B; S5 Table). Staurosporine (a positive control for the induction of apoptosis) increased the activation of caspases-3/7 by 199±19%. Incubation with Aβ40, which only slightly increased the number of PI-permeable cells, had no effect on caspase activity (103±14%). We can conclude that Aβ42, but not Aβ40, induced apoptotic cell death.

### Aβ peptides induce pathological changes in neurite morphology

**Fig 3:**
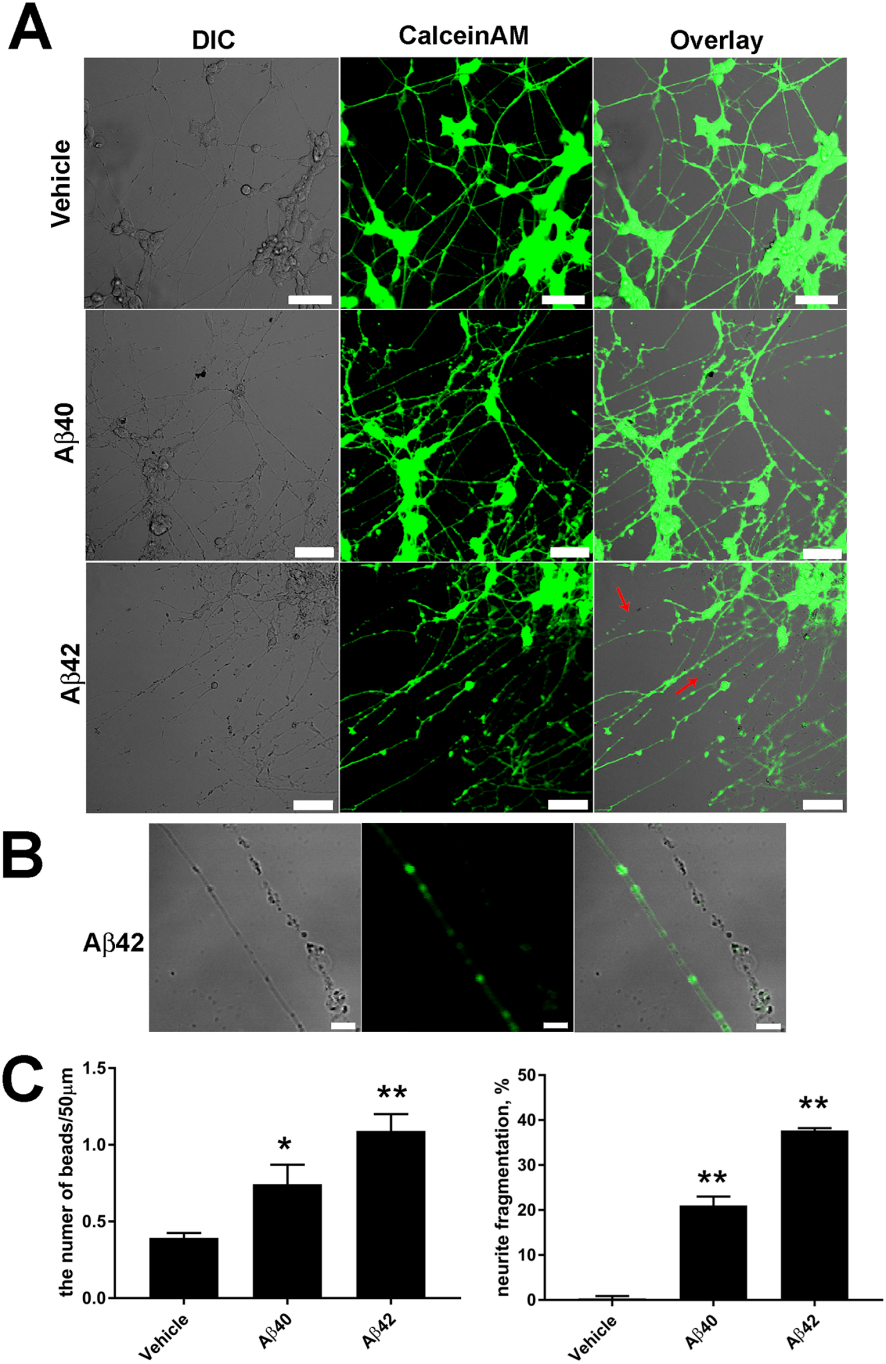
Aβ40 and Aβ42 induce pathological changes in neurite morphology after 72 h. (A) RA/BDNF differentiated live cell imaging for differential interference contrast (right), CalceinAM fluorescence (green; middle). Red arrows indicate fragmented neurites. Scale bar 20μm. (B) Greater magnification of a neurite with beads *versus* a fragmented neurite. Scale bar 5 μm. (C) Quantification of pathological changes in RA/BDNF differentiated cell culture. The figure displays the mean± SEM; at least *n = 3* independent experiments; **p≤0.005; *p≤0.05, One-way ANOVA followed by a Bonferroni's multiple comparisons test at the 0.05 level was used to determine differences between the conditions.

For the detection of Aβ-induced abnormalities in differentiated SH-SY5Y cells, the culture was stained with a CalceinAM dye that reveals viable cells and their extensions [33]. The morphological changes in neurites after incubation with Aβ peptides (Fig 3A) appeared considerably earlier than the Aβ mediated decrease in cell viability (Fig 2A). Aβ peptides induced beading and the subsequent fragmentation of neurites (Fig 3A, red arrows), whereas no CalceinAM signal was detected in fragmented neurites (Fig 3B). Quantitative analysis of the morphological changes in neurites showed that both peptides induced beading and fragmentation, but the influence of Aβ42 were considerably stronger than that of Aβ40 (Fig 3C; S6 and S7 Tables). The analysis of the early signs of microtubule disruption indicated that Aβ42 significantly induced bead formation. The amount of beads in the presence of Aβ42 per 50μm of neurite length increased to 1.12±0.08 compared to the vehicle 0.40 ± 0.03. Aβ40 did not induce statistically significant beading: 0.78 ± 0.11 beads per 50μm neurite length. Both peptides caused a substantial increase in the neurite fragmentation: 22.4±1.0% and 37.6±0.4% of neurites were fragmented in the presence of Aβ40 and Aβ42 respectively, whereas the fragmentation was not significant (0.57±0.57%) in the absence of the peptides.

### Aβ42 aggregates cover cell bodies and neurites

**Fig 4:**
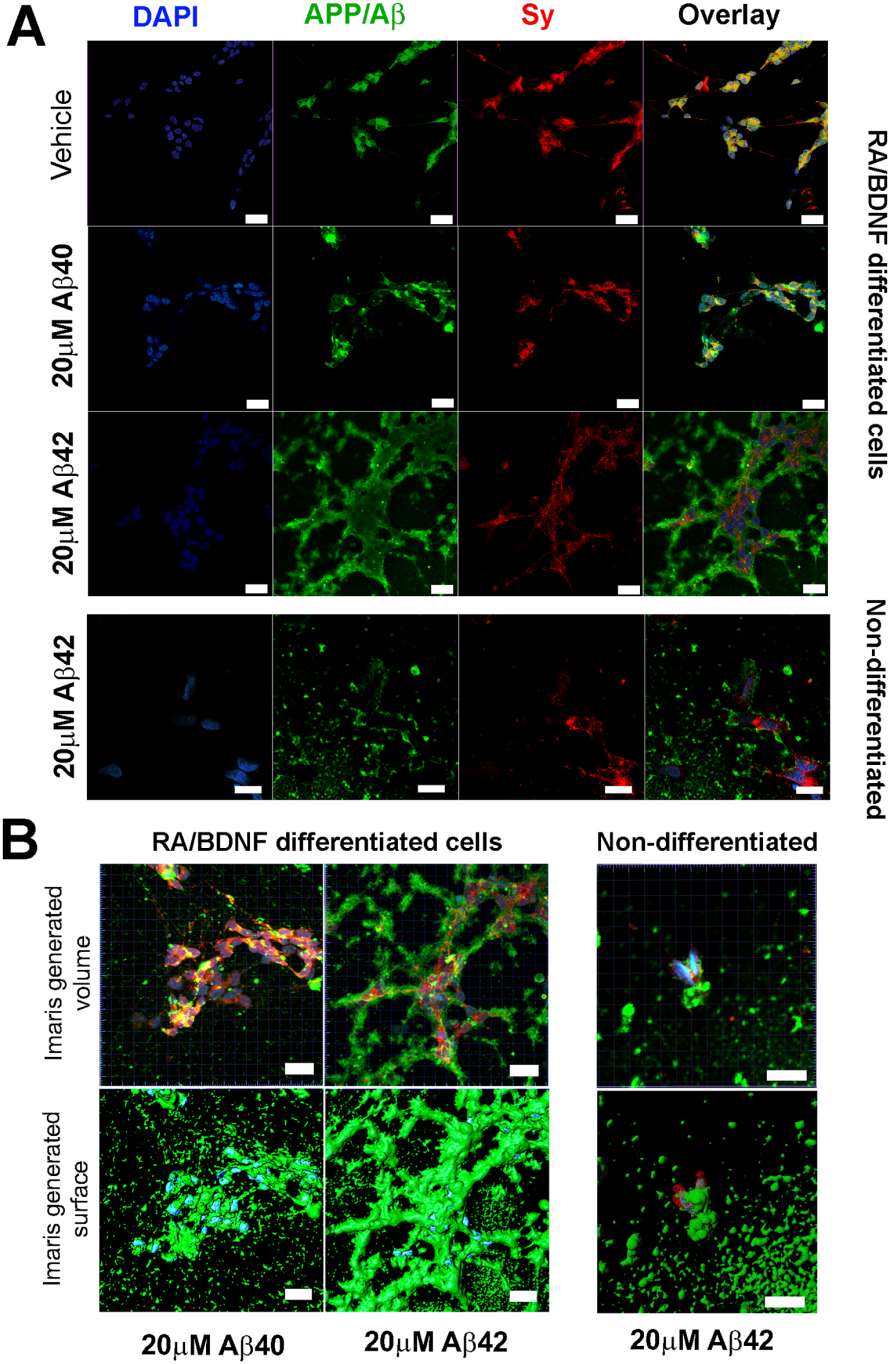
Aβ42 covers cell bodies and neurites. (A) Immunocytochemistry of non-differentiated and RA/BDNF differentiated SH-SY5Y cells after a 24h (Aβ42) and a 48h incubation (Vehicle; Aβ40) for DAPI (blue; left), anti-APP/Aβ (green; left middle), anti-Synaptophysin1 (red; right middle). Scale bar 20μm. (B) Three-dimensional reconstruction of differentiated cells incubated with 20μM Aβ40 and Aβ42 for 48h, and non-differentiated cells incubated with 20μM Aβ42 for 24h, stained for DAPI, anti-APP/Aβ, anti-Synaptophysin1. Confocal microscope images were obtained with optical section separation (z-interval) of 0.303 μm. Upper panels represent the virtually generated volume of chromophores and the lower panels represent the surface. Scale bar 20μm.

Since highly amyloidogenic Aβ-peptides can form fibrils on the cell surface in cell cultures [34–36], we studied the distribution of Aβ40 and Aβ42 using an anti-APP/Aβ antibody (Fig 4A). Panels presenting “Vehicle” demonstrate the endogenous APP/Aβ pattern in RA/BDNF differentiated cells. When cells were incubated with 20 μM peptide (lower panels) the presence of large extracellular Aβ aggregates appeared, which almost overshadowed the endogenous signals in the case of Aβ42. Three-dimensional reconstruction (Fig 4B) demonstrated that the cell bodies and neurites were fully covered with Aβ42 aggregates after 24 hours of incubation (Fig 4B, medium panels), whereas the less amyloidogenic Aβ40 did not cover the neurites and only random aggregates were present near and on the cell bodies after 48h (Fig 4B, right panels). Furthermore, this is in accordance with the fact that only Aβ42 induces caspase3/7 activation (Fig 2C). It could also be concluded that covering of neurites does not cause rapid cell death since a significant decrease in viability was observed only after incubation for 72 hours (Fig 2A). The non-differentiated cells were only partially covered with Aβ42 after 24h (Fig 4B, left panels). These results indicate that the Aβ42-induced toxic effects are related to the “Aβ cover” of neurites, because the non-differentiated cells lack long neuron-like extensions.

The presence of fibrillar aggregates in the cell medium containing Aβ42 after 48 hours of incubation was confirmed by TEM (S6 Fig), whereas no fibrils were detected in the medium containing Aβ40 under similar conditions. The fibrils formed in the cell culture were comparable to those previously generated in a test tube using peptides obtained from the same source [37]. Thioflavin T test showed that the aggregation of Aβ42 in serum free cell medium was completed within 24h (S7 Fig). In contrast to the significant toxic effect of Aβ42 fibrillizing in the solution surrounding the cells, the matured Aβ42 fibrils (preformed according to the protocol in Ref [38]) added to the culture medium had no toxic effect on the differentiated SH-SY5Y cells (S8 Fig). These data support the hypothesis that the toxic amyloid species may form during the fibrillization process [39].

## Discussion

During the last decades, when the amyloid hypothesis has been the prevailing concept in AD, research scientists have used various cellular and animal models to establish the toxic effects of Aβ-peptides and to search for possible “antidotes” [18, 40, 41]. Despite multiple promising candidates proposed as the result of these studies, none of the drug candidates have proved to be useful for AD patients. Although multiple examples of high Aβ toxicity can be found in the literature [15, 42–44], the toxicity of biologically more common peptide forms, e.g., monomers and fibrils, is often too low for use in test systems. The high toxicity values appear only under particular experimental conditions and are sometimes observed only when using particular test methods. For instance, Aβ peptides show high toxicity when cell viability is estimated by the MTT test. However, Aβ peptides have been shown to interfere with the MTT test, as the decrease in optical density is considerably larger than the decrease in the number of viable cells in the presence of Aβ [45–47]. Generating supersaturated Aβ solutions using organic solvents also enhance the peptide toxicity [21, 48, 49], however, this process has no analogue in living organisms. Aβ25–35 peptide, a fragment of Aβ consisting of amino acid residues only present in the non-amyloidogenic P3 peptide, also shows higher toxicity on cells than Aβ42. Aβ25–35 has not been found in the brains of AD patients and its high toxicity is related to the methionine in its C-terminal: the peptide variant with an additional amino acid in C-terminus is practically nontoxic under the same conditions [50]. Thus, Aβ25–35 is not a relevant substitution for Aβ-peptides in AD related studies [50]. It is desirable to use native peptides and biologically relevant procedures for the preparation of potentially toxic biomolecules in toxicological studies. Aβ peptides showed very low toxicity on non-differentiated SH-SY5Y cells, however the subsequent RA and BDNF treatment increased the susceptibility of the resulting neuron-like cells to the level applicable for toxicity studies and screening of putative protecting agents.

The most debatable topic in the amyloid hypothesis is which aggregation form of Aβ is toxic. Originally the amyloid fibrils were considered the neurotoxic species [23, 51]. Later on, interest concentrated on the more toxic Aβ oligomers generated by fast dilution of supersaturated Aβ solutions [22, 52]. Recently it has been proposed that the toxicity is related to the active fibrillization process: most likely the cells are affected by some metastable particles forming during the fibrillization [53]. In our experiments, only Aβ1–42 added in a predominantly monomeric form and fibrillizing in the cellular medium, induced apoptotic cell death. Both peptides Aβ1–40 and Aβ1–42 fibrillize within a half of an hour when the solution is vigorously agitated, whereas under quiescent conditions the fibrillization can take days to begin [54]. Once the fibrillization is initiated by adding fibrillary seeds or by agitation, Aβ1–42 retains a high fibrillization rate under non-agitated conditions, whereas the fibrillization rates of Aβ40 decrease substantially [55]. In our experiment, only Aβ42 that fibrillized within 24 hours in cellular medium was toxic for the cells, thus, relating the toxicity of amyloid to a myriad of dynamic intermediates present in the solution during the fibrillization. According to the relatively new concept that secondary nucleation mechanisms prevail in amyloidogenesis, the growing fibrils are partially converted to small amyloid species, which are considered to be toxic and also responsible for propagation of plaques [39]. Our results support the general idea of this hypothesis, because pre-formed mature fibrils were not toxic for the cells.

Matsuzaki et al. demonstrated that amyloid fibrils growing on cells cause membrane deformation [35]. In our experiments Aβ1–42, but not Aβ1–40, covered the cell neurites and cell bodies with a dense fibrillary coating, which lead to a significant increase in membrane permeability as demonstrated by the PI test. The mechanism of how the intermediate Aβ1–42 aggregates induce apoptosis is not clear, for instance, they could form pores in cell membranes [56]. It is important to note that the non-differentiated cells are also partially covered with Aβ1–42, however, their viability was not significantly affected. The non-differentiated cells lack long neurites that have an especially tight amyloid cover and have a smaller surface area, thus, their contact area with Aβ is substantially smaller. Recently it was demonstrated that small fibrillary aggregates of Aβ consisting of 50–70 monomers are always present in Aβ1–42, but not in Aβ1–40 solutions right after the dilution [55]. This data can be invaluable in understanding the peptide toxicity–only the longer peptide containing fibrillary seeds and capable of forming fibrils in the cell culture medium had a substantial toxic effect on the cells.

RA/BDNF-differentiated SH-SY5Y cells with neuron-like morphology were more susceptible to Aβ than the non-differentiated cells. We demonstrated that Aβ1–42 impairs neurites and this is followed by apoptotic cell death. Similar results were obtained with primary cultures [10, 11], supporting the suggestion that RA/BDNF-differentiated SH-SY5Y cells are an appropriate model for studying the effects of amyloids on neuronal cells. The peptides showed relatively low toxicity on the RA/BDNF-differentiated cells *in vitro*, which is in agreement with the slow progression of AD, however, a question may arise about the biological relevance of the effects observed at extremely high concentrations of these effectors. Considering that in the AD brain neurons nearby the plaques are dying [57], it is the contact with peptide aggregate and not the bulk peptide concentration that is the trigger for cell death. The high concentration used in cell experiments ensures that all cells are influenced by the toxic entity–growing fibrils. The main plaque component Aβ42 was more toxic on the differentiated SH-SY5Y cells than its less malignant counterpart Aβ40, most likely because of its stronger ability to form fibrils and cover neurites with a dense coating. Taken together, our findings show that amyloid fibrils, formed *in situ* in the cell culture, induce beading and neurite fragmentation in differentiated human neuron-like SH-SY5Y cells. The RA/BDNF- differentiated SH-SY5Y cells can be used in further detailed studies of the Aβ toxicity on neuronal cells and *in vitro* screening of putative drugs that suppress the Aβ toxicity [58, 59].

## Conclusion

The current study showed that the RA/BDNF-differentiated human SH-SY5Y cell line is substantially more sensitive to amyloid peptides than non-differentiated cells. Aβ42 fibrils forming spontaneously in the culture had clear toxic effect on the cells and caused neuritic abnormalities and caspase activation similar to the processes in the brain of patients with neurodegeneration, whereas Aβ40 was non-toxic. It can be concluded that the RA/BDNF- differentiated SH-SY5Y cells can serve as a reasonably good *in vitro* model for the study of neuronal death in AD. Further studies are needed to describe the putative mechanism of pathology in this model.

## Supporting information

S1 Fig Representative photograph of non-differentiated and RA/BDNF differentiated cells in phase contrast. (PDF)

S2 Fig Amyloid beta quality control by NMR, MALDI-MS and SDS-PAGE. (PDF)

S3 Fig Representative snapshot of the process of the evaluation of neurite degeneration. (PDF)

S4 Fig WST-1 test results on non-differentiated cells without serum. (PDF)

S5 Fig Representative photograph of RA-differentiated SH-SY5Y cells. (PDF)

S6 Fig Transmission electron microscopy of cell medium after a 48 h incubation with amyloid peptides. (PDF)

S7 Fig Detection of amyloid aggregation by Thioflavin T in the cell medium. (PDF)

S8 Fig Cell viability after a 72 h incubation with 20 μM previously formed fibrils measured with the WST-1 test and membrane integrity counted with the propidium iodide permeabilization tests. (PDF)

S1 Table Non-differentiated SH-SY5Y cells, cell viability WST-1 test. (PDF)

S2 Table Non-differentiated SH-SY5Y cells, propidium iodide test. (PDF)

S3 Table RA/BDNF-differentiated SH-SY5Y cells, cell viability WST-1 test. (PDF)

S4 Table RA/BDNF-differentiated SH-SY5Y cells, propidium iodide test. (PDF)

S5 Table Effect of Aβ42 on the activities of caspase-3 and/or 7 on RA/BDNF differentiated cells. (PDF)

S6 Table The number of beads per 50 μM of neurite length after 72 h with 20 μM peptide. (PDF)

S7 Table Proportion of fragmented neurites per area after 72 h with 20 μM peptide. (PDF)

## Abbreviations

AD: Alzheimer’s disease
APP: amyloid precursor protein
ATCC: American Type Culture Collection
Aβ: amyloid beta
BDNF: brain derived neurotrophic factor
CalceinAM: calcein-acetoxymethylester
DAPI: 4',6-diamidino-2-phenylindole
DIC: differential interference contrast
DMEM: Dulbecco's modified Eagle’s medium
DMSO: dimethyl sulfoxide
EDTA: Ethylenediaminetetraacetic acid
HEPES: 4-(2-hydroxyethyl)-1-piperazineethanesulfonic acid
HFIP: 1,1,1,3,3,3-hexafluoro-2-propanol
LSM: laser scanning microscope
MALDI-MS: matrix-assisted laser desorption ionization mass spectrometry
MTT: 3-(4,5-dimethylthiazol-2-yl)-2,5-diphenyltetrazolium bromide
RA: retinoic acid
ROS: reactive oxygen species
SDS-PAGE: sodium dodecyl sulfate polyacrylamide gel electrophoresis
Sy: Synaptophysin1
TUJ-1: beta III tubulin
WST: water soluble tetrazolium
