# Supplementary material for: In situ fibrillizing amyloid-beta 1-42 induces neurite degeneration and apoptosis of differentiated SH-SY5Y cells
Source: PLoS One. 2017 Oct 24;12(10):e0186636. doi: 10.1371/journal.pone.0186636 (PMC5655426; doi:10.1371/journal.pone.0186636)
Supplement: S1 Fig — (PDF) [file pone.0186636.s001.pdf]

S1 Fig.

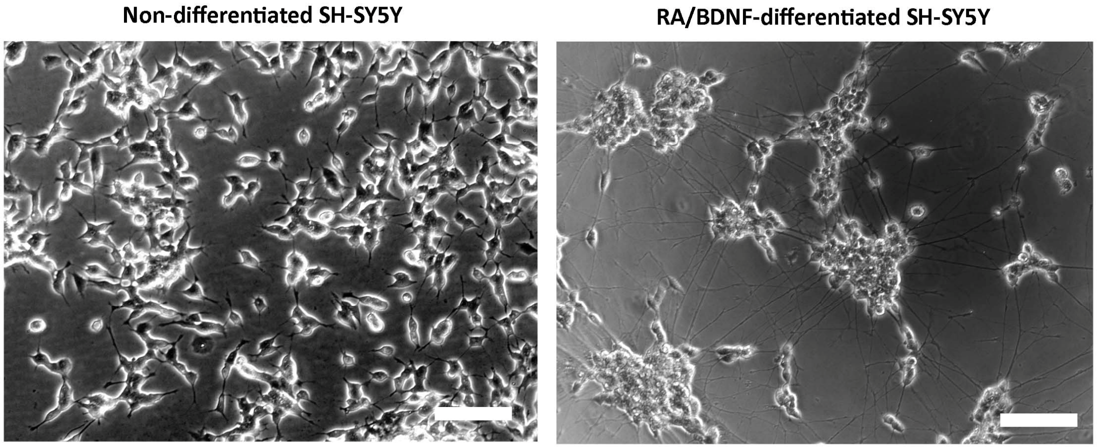

S1 Fig. Representative photograph of non-differentiated and RA/BDNF differentiated cells in phase contrast[1]. Scale bar 100µM.

[1] Images obtained with Zeiss Axiovert 200M, 20x objective.
