# Supplementary material for: In situ fibrillizing amyloid-beta 1-42 induces neurite degeneration and apoptosis of differentiated SH-SY5Y cells
Source: PLoS One. 2017 Oct 24;12(10):e0186636. doi: 10.1371/journal.pone.0186636 (PMC5655426; doi:10.1371/journal.pone.0186636)
Supplement: S2 Fig — (PDF) [file pone.0186636.s002.pdf]

## S2 Fig.

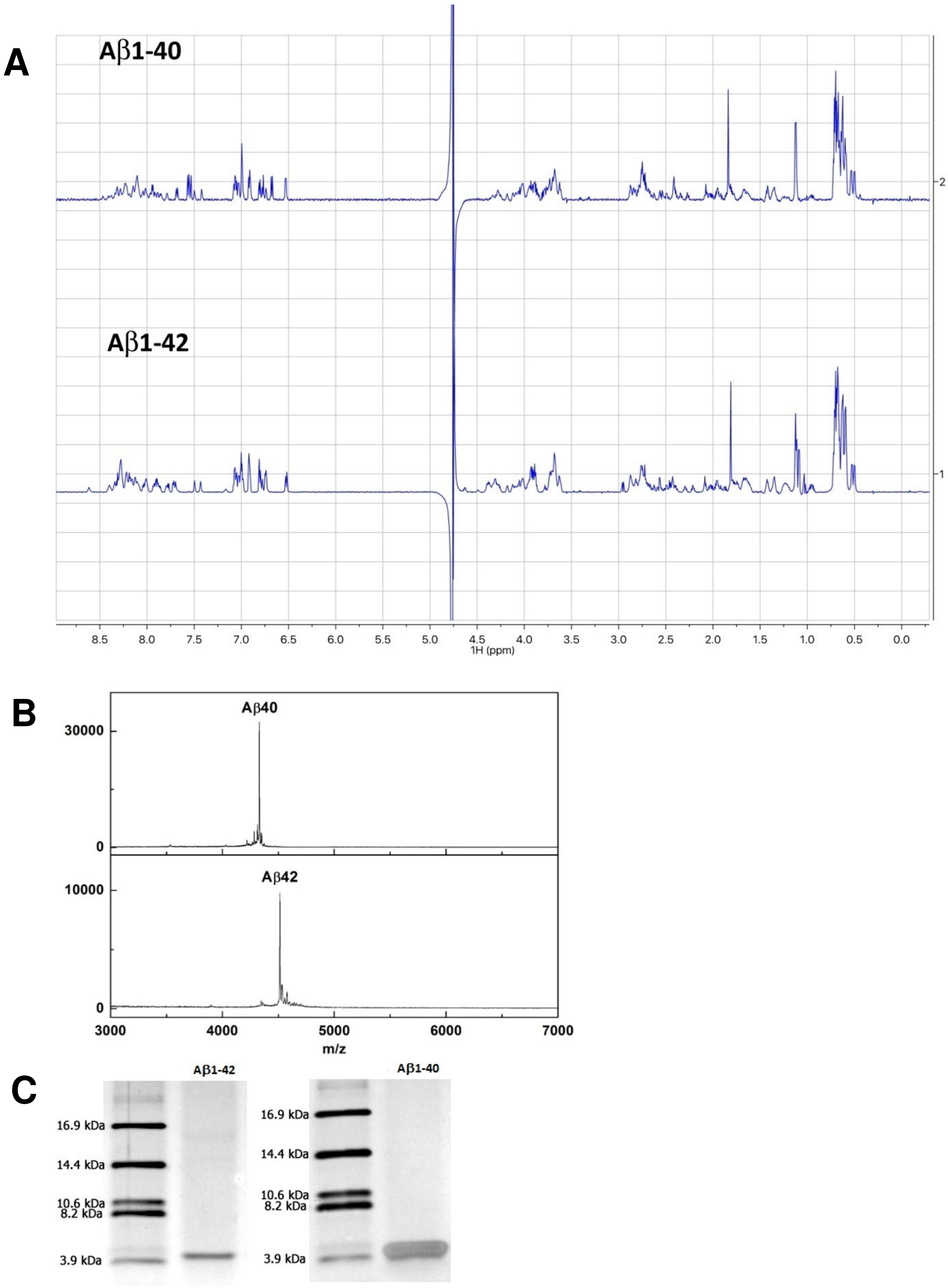
A
Aβ1-40
Aβ1-42
8.5 8.0 7.5 7.0 6.5 6.0 5.5 5.0 4.5 4.0 3.5 3.0 2.5 2.0 1.5 1.0 0.5 0.0
1H (ppm)
2
1
B
Aβ40
30000
0
Aβ42
10000
0
3000 4000 5000 6000 7000
m/z
C
Aβ1-42
16.9 kDa
14.4 kDa
10.6 kDa
8.2 kDa
3.9 kDa
Aβ1-40
16.9 kDa
14.4 kDa
10.6 kDa
8.2 kDa
3.9 kDa

**S2 Fig. Amyloid beta quality control: NMR[2] spectra (A); MALDI-MS[3] (B); SDS-PAGE[4] (C)**

[2] Nuclear magnetic resonance (NMR) spectra were recorded at 278K and pH 7.4 in a Shigemi 5mm PMS3 tube with the spectrometer frequency of 800.13MHz. The length of the calibrated 90° pulse was set to8.9 $\mu$s (the total time of measurment was 7 min with the number of scans at 128) . The sample contained peptide at a concentration of 70 $\mu$M and was buffered with 50mM potassium phosphate and 5mM sodium hydroxichloride. The NMR experiments were performed on an Avance III 800 MHz spectrometer (Bruker, Karlsruhe, Germany), equipped with the 5-mm standard probe TXI 800 MHz S4 with Z-gradient.

[3] Matrix-assisted laser desorption mass spectrometry. Spectra were acquired by *Voyager-DE™ STR Biospectrometry Workstation* in linear mode using automated program. Instrument parameter/settings: accelerating voltage 25 000 V; mass range (m/z) 1500-10 000 Da; delay time 485 ns; grid voltage 93%; laser intensity 2200 V

[4] Sodium dodecyl sulfate poly acrylamide gel electrophoresis (SDS-PAGE) was performed using *Mini-PROTEAN TetraSystem* (Bio-Rad). Samples were mixed with loading buffer (0.36 M Bistris, 0.053 M Bicine, 15% glycerol, 1% SDS, 0.004% bromophenol blue), maintained at room temperature, applied to Bicine-Tris 15%T/5%C gel and resolved in a cathode buffer with 0.25% SDS (110V). Gels were fixated in glutar aldehyde/borate buffer solution for 45 minutes and stained with silver according to a protocol in Ref 1. Dunn, M.J. and S.J. Crisp, *Detection of proteins in polyacrylamide gels using an ultrasensitive silver staining technique.* Methods Mol Biol, 1994. **32**: p. 113-8.
