# Supplementary material for: In situ fibrillizing amyloid-beta 1-42 induces neurite degeneration and apoptosis of differentiated SH-SY5Y cells
Source: PLoS One. 2017 Oct 24;12(10):e0186636. doi: 10.1371/journal.pone.0186636 (PMC5655426; doi:10.1371/journal.pone.0186636)
Supplement: S3 Fig — (PDF) [file pone.0186636.s003.pdf]

S3 Fig.

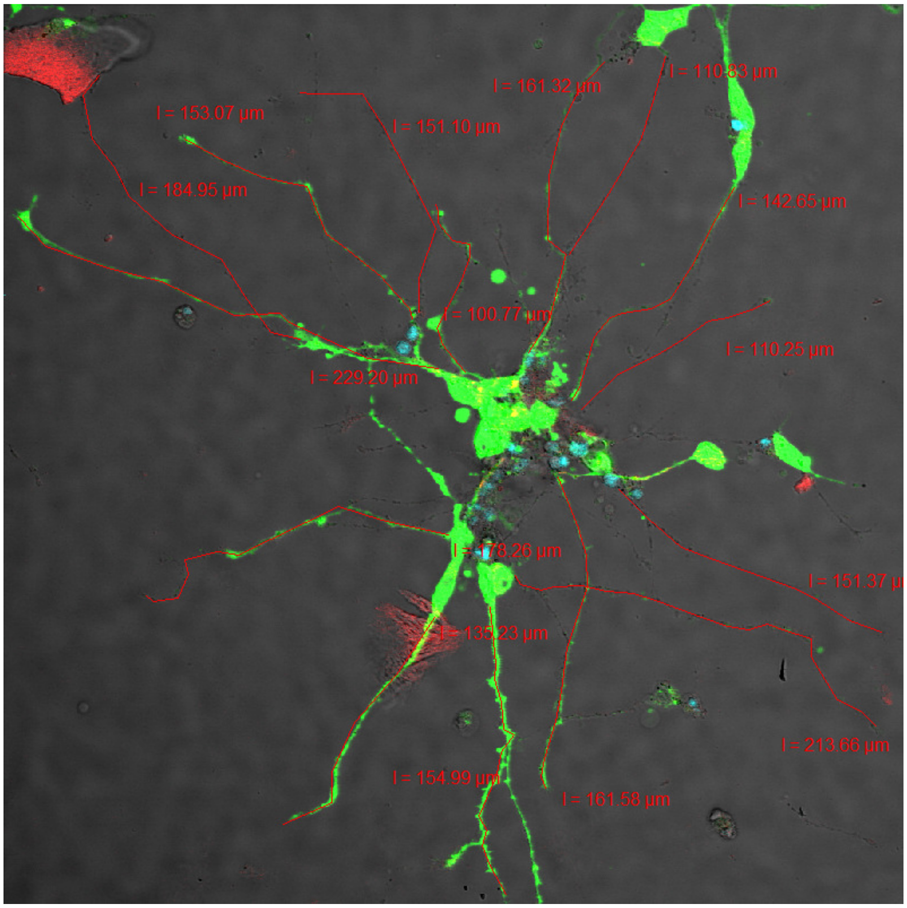

S3 Fig. Representative snapshot of the process of the evaluation of neurite degeneration[4]. The image is the overlay of three channels: DIC, Calcein AM (green), DAPI (blue).

---

[4] Photomicrographs of at least 12 random areas of neurites were taken using Zeiss Duo 510 META with 20X objective and 63X objective with oil immersion. Neurites were stained with CalceinAM (calcein-acetoxymethyl ester) for the analysis. General morphology of neurites was obtained with differential interference contrast (aperture DIC2) technique. The microscopy experiments were performed in an incubation chamber at 37^0C in the presence of 5% CO_2.

Photomicrographs of neurites in randomly selected areas were stored and processed using LSM Image Browser software. The method of evaluation of neurite degeneration was adopted from Ref. 2. Kawataki, T., et al., *Neuronal maturation-associated resistance of neurite degeneration caused by trophic factor deprivation or microtubule-disrupting agents.* Brain Res, 2008. **1230**: p. 37-49. The number of beads per total length of measured neurites was counted. Medium or thin neurites in captured regions were chosen for this purpose. The number of beadings/50 µm length was counted and averaged over at least 8 neurites for each area. The proportion of fragmented neurites was counted for each area and expressed as a percentage of the total amount of counted neurites longer than 100 µm.
