# Supplementary material for: In situ fibrillizing amyloid-beta 1-42 induces neurite degeneration and apoptosis of differentiated SH-SY5Y cells
Source: PLoS One. 2017 Oct 24;12(10):e0186636. doi: 10.1371/journal.pone.0186636 (PMC5655426; doi:10.1371/journal.pone.0186636)
Supplement: S4 Fig — (PDF) [file pone.0186636.s004.pdf]

S4 Fig.

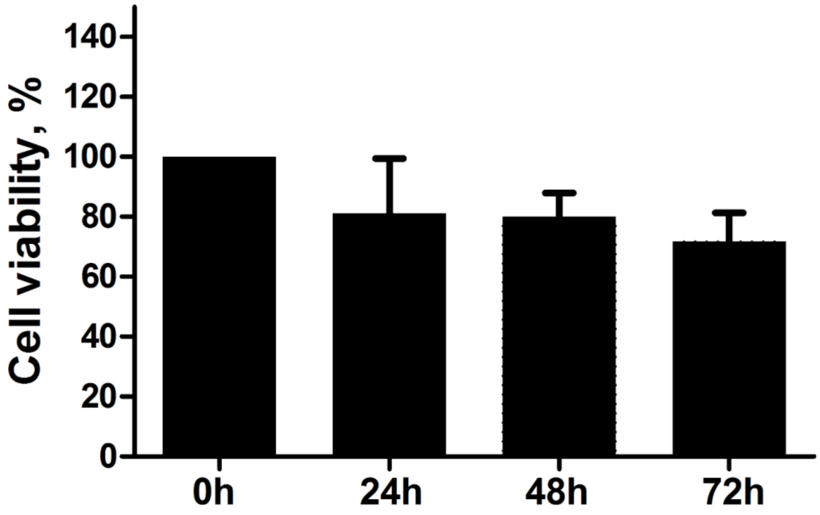

S4 Fig. WST-1 test results on non-differentiated cells without serum (See Materials and Methods). Data are shown as mean± SEM
