# Supplementary material for: In situ fibrillizing amyloid-beta 1-42 induces neurite degeneration and apoptosis of differentiated SH-SY5Y cells
Source: PLoS One. 2017 Oct 24;12(10):e0186636. doi: 10.1371/journal.pone.0186636 (PMC5655426; doi:10.1371/journal.pone.0186636)
Supplement: S5 Fig — (PDF) [file pone.0186636.s005.pdf]

S5 Fig.

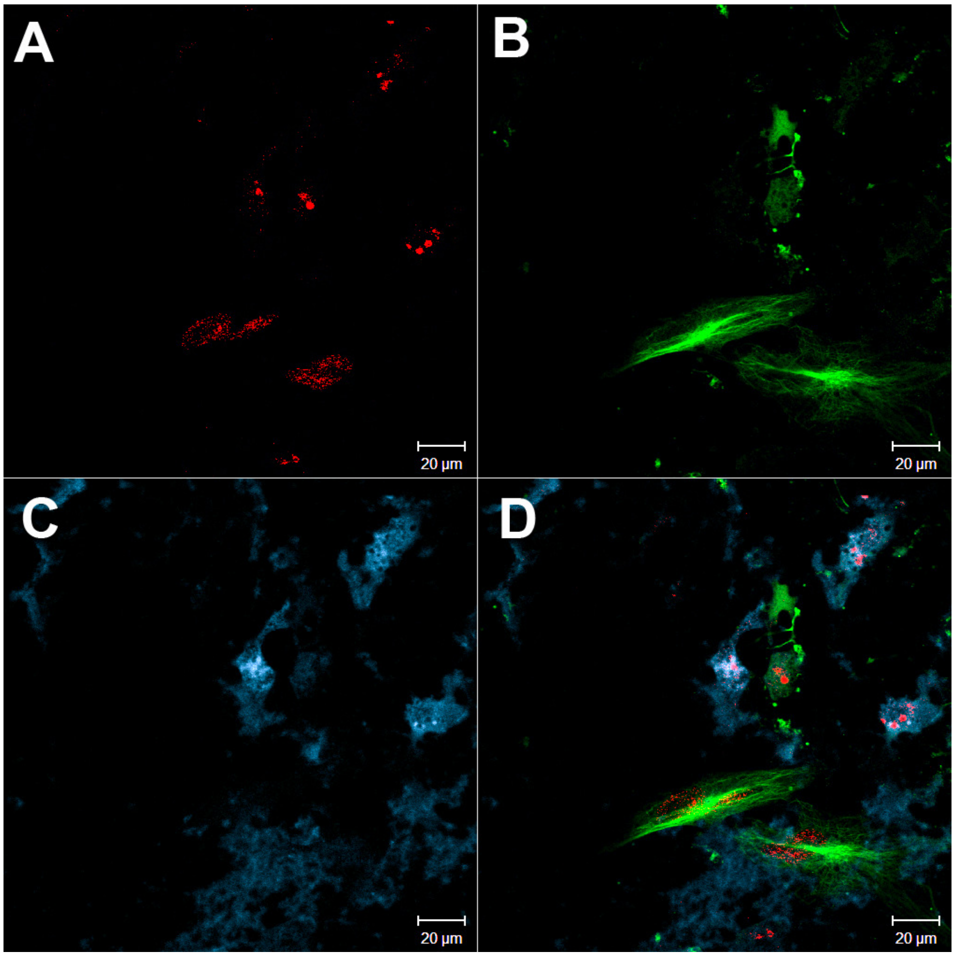

S5 Fig. Representative photograph of RA-differentiated SH-SY5Y cells after incubation with 20µM Aβ42. *In case of RA differentiation cell culture is not enough homogenic. In the photograph several cells are not amyloid beta-affected: microtubules are intact. At the same time, nuclear fragmentation and microtubule disruption can be detected.* (A) Nuclei staining with PI[5] (red) showing nuclear fragmentation (B) βIII-tubulin staining (green) showing different cell types, (C) Fluorescamine-stained Aβ42[6] (blue), (D) merged image. Magnification: 630X, scale bar: 20µm

[5] Methanol-fixed cells were stained with propidium iodide/RNAse solution for 10 min.

[6] Peptides were stained with fluorescamine (FC, Sigma) for microscopy. FC is a non-fluorescent dye that reacts with primary amines to form a fluorescent product. Defibrillized peptides were dissolved in 10 mM NaOH and then FC stock in acetonitrile (Sigma) was added in 2-fold higher concentration than peptide concentration. The mixture was incubated for 10 min at RT in dark and afterwards diluted in 40 mM HEPES/200 mM NaCl buffer (pH 7,3) for suitable concentration and applied on cell culture.
