# Supplementary material for: In situ fibrillizing amyloid-beta 1-42 induces neurite degeneration and apoptosis of differentiated SH-SY5Y cells
Source: PLoS One. 2017 Oct 24;12(10):e0186636. doi: 10.1371/journal.pone.0186636 (PMC5655426; doi:10.1371/journal.pone.0186636)
Supplement: S6 Fig — (PDF) [file pone.0186636.s006.pdf]

S6 Fig.

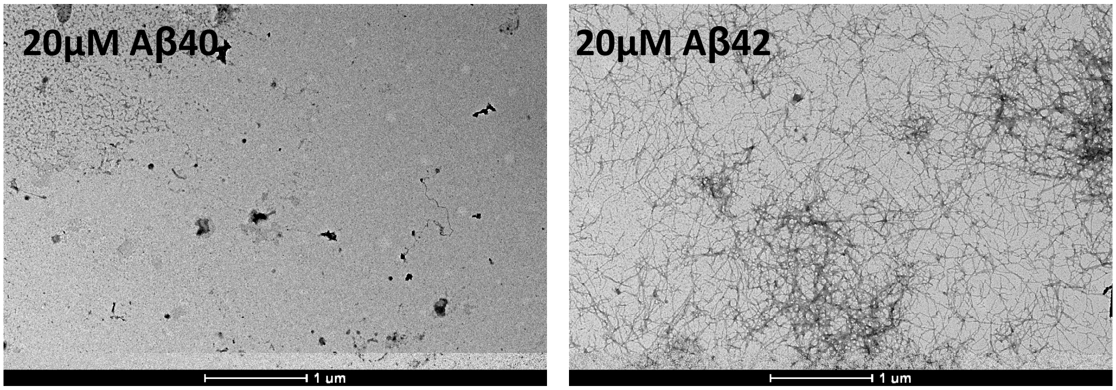

S6 Fig. Transmission electron microscopy[7] of cell medium after a 48 h incubation with amyloid peptides. Scale bar 1 μm.

7 Transmission Electron Microscopy Imaging An aliquot of 5 μl of sample was loaded on a Formvar-coated, carbon-stabilized copper grid (300 mesh from Ted Pella Inc., Redding CA, USA). After 1 min, the excess solution was drained off using a Whatman filter paper (Thermo Fisher). The grid was briefly washed and negatively stained with 5 μl of 2% uranyl acetate. The grid was air-dried and the transmission electron microscopy (TEM) images were recorded on a Tecnai G2 BioTwin transmission electron microscope (FEI, Japan) operating with an accelerator voltage of 120 kV, 1.53 nA. Typical magnifications ranged from 4800x to 18 500x.
