# Supplementary material for: In situ fibrillizing amyloid-beta 1-42 induces neurite degeneration and apoptosis of differentiated SH-SY5Y cells
Source: PLoS One. 2017 Oct 24;12(10):e0186636. doi: 10.1371/journal.pone.0186636 (PMC5655426; doi:10.1371/journal.pone.0186636)
Supplement: S7 Fig — (PDF) [file pone.0186636.s007.pdf]

S7 Fig.

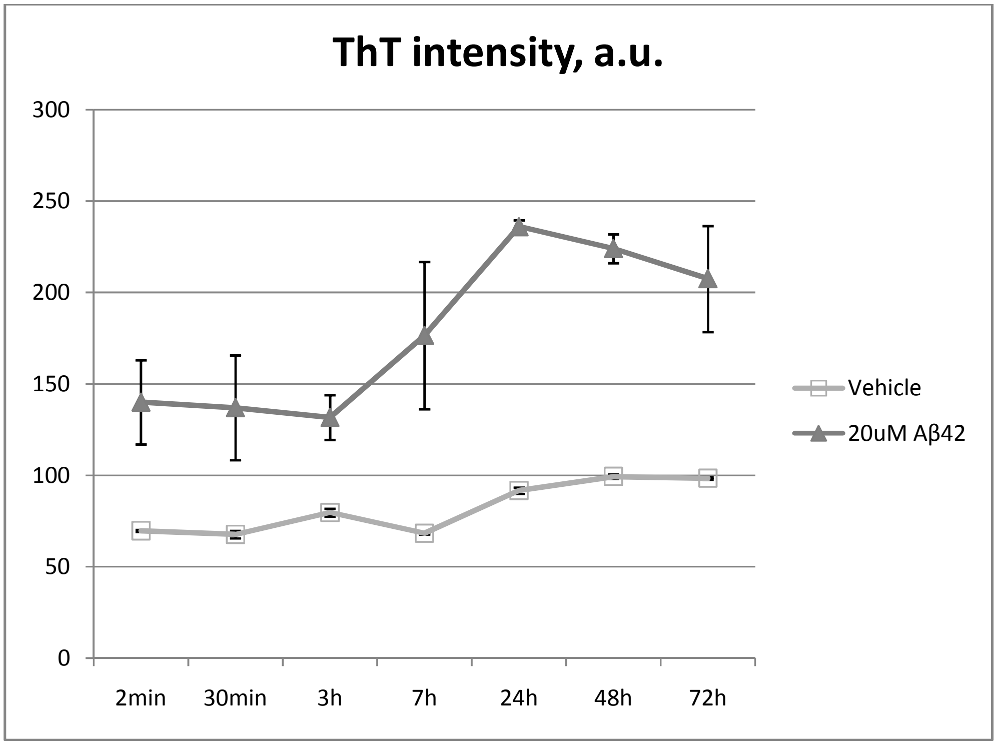

S7 Fig. Detection of amyloid aggregation by Thioflavin T in the cell medium[8]. Vehicle is the HEPES buffer without peptide (See Materials and Methods). The error bars are displayed with ±SD; n=3

[8] Aβ stock mixtures (See materials and methods) were diluted in DMEM without serum to a final peptide concentration of 20 μM and 5 μM Thioflavin T was added. The white 96 well plate was used for the experiments and incubated in the cell incubator at 37°C 5% CO2. The fluorescence of binded ThT were measured by a Tecan Microplates reader, series 750 (ex 405nm em 485nm).
