# Supplementary material for: In situ fibrillizing amyloid-beta 1-42 induces neurite degeneration and apoptosis of differentiated SH-SY5Y cells
Source: PLoS One. 2017 Oct 24;12(10):e0186636. doi: 10.1371/journal.pone.0186636 (PMC5655426; doi:10.1371/journal.pone.0186636)
Supplement: S8 Fig — (PDF) [file pone.0186636.s008.pdf]

S8 Fig.

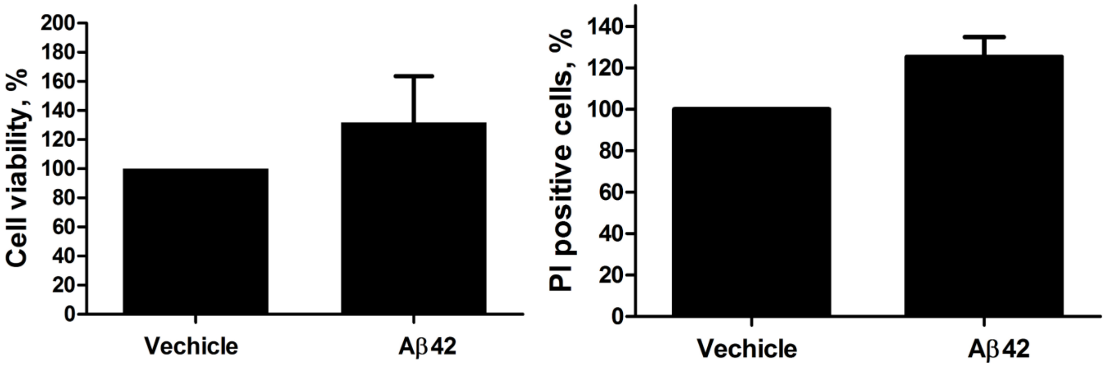

S8 Fig. Cell viability after a 72h incubation with 20μM previously formed fibrils[9] measured with the WST-1 test and membrane integrity counted with the propidium iodide permeabilization tests (See Materials and Methods). Data are shown with the mean± SEM, n=3.

[9] The fibrills were performed according to the protocol in Ref 3. Tiiman, A., et al., *In vitro fibrillization of Alzheimer's amyloid-beta peptide (1-42).* AIP Advances, 2015. **5**(9): p. 092401. After fibrillization detection, the solutions were collected and stored overnight at room temperature, then centrifuged at 10 000rcf for 10min at 4°C. The pellets were resuspended in the buffer (See Materials and Methods) and applied on cells.
