# Supplementary material for: In situ fibrillizing amyloid-beta 1-42 induces neurite degeneration and apoptosis of differentiated SH-SY5Y cells
Source: PLoS One. 2017 Oct 24;12(10):e0186636. doi: 10.1371/journal.pone.0186636 (PMC5655426; doi:10.1371/journal.pone.0186636)
Supplement: S1 Table — (PDF) [file pone.0186636.s009.pdf]

S1 Table: Non-differentiated SH-SY5Y cells, cell viability WST-1 test.

	48h		72h	
Vehicle	Aβ40	Aβ42	Aβ40	Aβ42
100%	112.7	83.9	96.9	96.7
	114.8	88.9	99.8	111.1
	106.1	91.2	91.2	83.7
	111.0	70.6	112.1	
	109.1		106.8	
Average	110.7	83.6	101.4	97.1
SEM	1.5	4.6	3.7	7.9
