# Supplementary material for: In situ fibrillizing amyloid-beta 1-42 induces neurite degeneration and apoptosis of differentiated SH-SY5Y cells
Source: PLoS One. 2017 Oct 24;12(10):e0186636. doi: 10.1371/journal.pone.0186636 (PMC5655426; doi:10.1371/journal.pone.0186636)
Supplement: S2 Table — (PDF) [file pone.0186636.s010.pdf]

S2 Table: Non-differentiated SH-SY5Y cells, propidium iodide test.

	48h		72h	
Vehicle	Aβ40	Aβ42	Aβ40	Aβ42
100%	118.2	101.2	154.5	119.5
	95.0	117.1	102.0	133.1
	110.4	96.3	116.6	138.3
		117.1		
		125.1		
Average	107.9	111.4	124.4	130.3
SEM	6.8	5.4	15.6	5.6
