# Supplementary material for: In situ fibrillizing amyloid-beta 1-42 induces neurite degeneration and apoptosis of differentiated SH-SY5Y cells
Source: PLoS One. 2017 Oct 24;12(10):e0186636. doi: 10.1371/journal.pone.0186636 (PMC5655426; doi:10.1371/journal.pone.0186636)
Supplement: S3 Table — (PDF) [file pone.0186636.s011.pdf]

S3 Table: RA/BDNF-differentiated SH-SY5Y cells, cell viability WST-1 test.

	48h		72h	
Vehicle	Aβ40	Aβ42	Aβ40	Aβ42
100%	89.8	81.5	67.1	54.9
	82.0	109.4	77.0	57.4
	71.7	38.4	66.2	59.6
		69.0		
		80.2		
		86.9		
Average	81.2	77.6	70.1	57.3
SEM	4.6	9.5	3.4	1.3
