# Supplementary material for: In situ fibrillizing amyloid-beta 1-42 induces neurite degeneration and apoptosis of differentiated SH-SY5Y cells
Source: PLoS One. 2017 Oct 24;12(10):e0186636. doi: 10.1371/journal.pone.0186636 (PMC5655426; doi:10.1371/journal.pone.0186636)
Supplement: S4 Table — (PDF) [file pone.0186636.s012.pdf]

S4 Table: RA/BDNF-differentiated SH-SY5Y cells, propidium iodide test.

	48h		72h	
Vehicle	Aβ40	Aβ42	Aβ40	Aβ42
100%	97.7	137.5	97.9	154.2
	98.5	183.9	133.3	158.4
	97.1	100.0	157.3	138.6
		119.4	127.9	
		112.8		
Average	97.7	130.7	129.1	150.4
SEM	0.4	14.6	12.2	6.0
