# Supplementary material for: In situ fibrillizing amyloid-beta 1-42 induces neurite degeneration and apoptosis of differentiated SH-SY5Y cells
Source: PLoS One. 2017 Oct 24;12(10):e0186636. doi: 10.1371/journal.pone.0186636 (PMC5655426; doi:10.1371/journal.pone.0186636)
Supplement: S5 Table — (PDF) [file pone.0186636.s013.pdf]

S5 Table: Effect of Aβ42 on the activities of caspase-3 and/or 7 on RA/BDNF differentiated cells.

Vehicle	Aβ40	Aβ42	Staur
100%	90.4	146.8	192.0
	110.7	175.0	227.1
	109.8	173.7	202.2
	117.8	158.6	198.1
	84.1	146.9	203.1
			169.7
Average	102.6	160.2	198.7
SD	14.5	13.8	18.6
