# Supplementary material for: In situ fibrillizing amyloid-beta 1-42 induces neurite degeneration and apoptosis of differentiated SH-SY5Y cells
Source: PLoS One. 2017 Oct 24;12(10):e0186636. doi: 10.1371/journal.pone.0186636 (PMC5655426; doi:10.1371/journal.pone.0186636)
Supplement: S6 Table — (PDF) [file pone.0186636.s014.pdf]

S6 Table: The number of beads per 50μM of neurite length after 72h with 20μM peptide.

	Vehicle	Aβ40	Aβ42
	0.4	0.9	1.2
	0.4	0.7	1.0
	0.4	0.7	1.1
Average	0.4	0.8	1.1
SEM	0.0	0.1	0.1
