# Supplementary material for: In situ fibrillizing amyloid-beta 1-42 induces neurite degeneration and apoptosis of differentiated SH-SY5Y cells
Source: PLoS One. 2017 Oct 24;12(10):e0186636. doi: 10.1371/journal.pone.0186636 (PMC5655426; doi:10.1371/journal.pone.0186636)
Supplement: S7 Table — (PDF) [file pone.0186636.s015.pdf]

S7 Table: Proportion of fragmented neurites per area after 72h with 20μM peptide.

	Vehicle	Aβ40	Aβ42
	1	23	38
	0	21	37
	0	19	38
Average	0.6	22.4	37.6
SEM	0.6	1.0	0.4
